# Chronic Venous Insufficiency: Transforming Growth Factor-β Isoforms and Soluble Endoglin Concentration in Different States of Wound Healing

**DOI:** 10.3390/ijms18102206

**Published:** 2017-10-21

**Authors:** Daniela Ligi, Lidia Croce, Giovanni Mosti, Joseph D. Raffetto, Ferdinando Mannello

**Affiliations:** 1 Department of Biomolecular Sciences, Section of Clinical Biochemistry and Molecular Genetics, University “Carlo Bo”, 61029 Urbino, Italy; daniela.ligi@uniurb.it (D.L.); lidia.croce@uniurb.it (L.C.); 2 Department of Angiology, Barbantini Clinic, via del Calcio 2, 55100 Lucca, Italy; giovanni.mosti10@gmail.com; 3 Vascular Surgery Division, VA Boston Healthcare System, West Roxbury, Boston, MA 02130, USA; joseph.raffetto@va.gov; 4 Harvard Medical School, Brigham and Women’s Hospital, Boston, MA 02115, USA

**Keywords:** chronic venous insufficiency, inflammation, glycosaminoglycan, sulodexide, venous leg ulcer, wound healing, soluble endoglin, transforming growth factor beta isoforms, monocyte

## Abstract

Venous leg ulcer (VLU) is a huge healthcare problem with poorly understood pathophysiology. Transforming growth factor-β (TGF-β) and endoglin (Eng), are inflammatory and wound healing mediators. Eng, co-receptor for TGF-β type-II receptors, may be cleaved forming soluble Eng (sEng), antagonizing TGF-β signaling, a crucial process in vascular pathologies. We evaluated the accumulation in wound fluid (WF) of TGF-β isoforms and sEng in healing stages, showing the effects of sulodexide treatments, a glycosaminoglycan with clinical efficacy in VLU healing. Patients with inflammatory (Infl) and granulating (Gran) VLU were recruited. WFs and THP-1 monocytes exposed to Infl and Gran WF (treated/untreated with sulodexide) were analyzed for TGF-β isoforms and sEng by multiplex immunoassay. In both Infl and Gran WF, TGF-β1 and β2 were similar; TGF-β3 was significantly increased in Infl compared to Gran WFs (*p* = 0.033). sEng was significantly elevated in Gran compared to Infl WFs (*p* = 0.002). In THP-1 monocytes there was a significant increase in sEng after co-treatment of WF and sulodexide. The increase in TGF-β3 found in Infl WF highlights its negative effect on wound healing, while the increased levels of sEng in Gran WF affects the leukocyte adhesion/transmigration through the endothelium, reducing the inflammatory response and favoring the wound healing. Glycosaminoglycan sulodexide potentiates the effects of sEng release from monocyte, representing an important therapeutic option for wound healing.

## 1. Introduction

Venous leg ulcer (VLU) is the final complication of chronic venous disease (CVeD), a common lower extremity vascular pathology of great medical and socioeconomic impact, affecting a large part of the population worldwide [1]. Although several studies have been focused to identify biomolecular pathways of healing/nonhealing processes, the complex pathogenetic mechanisms and the characterization of VLU microenvironment are not fully understood [2]. So, there is an urgent need to find diagnostic/prognostic biochemical tests for early identification of hard-to-heal wounds, and allowing a more effective targeted therapy [3].

In general, wound healing is a complex process dependent on epithelialization, the formation of granulation tissue, the remodeling of the extracellular matrix (ECM), and scar formation [1]. In particular, whereas normal healing of acute wounds is traditionally divided into four overlapping and timely-limited phases (i.e., hemostasis, inflammation, granulation, and remodeling), chronic wounds seem to be blocked mainly in a persistent inflammatory state, which prevents the progression toward the next phases and finally the wound closure [2]. In this regard, “non-healing ulcers are wounds which do not show any reduction in size within six months”; this definition includes both ulcers blocked in the inflammatory phase of wound healing (inflammatory ulcers), and ulcers which, due to conservative or surgical therapeutical procedures, turned granulation phase but did not start to reduce in size (granulating ulcers) [4,5].

However, it remains unclear why some chronic leg ulcers heal whereas others are recalcitrant. Therefore, the study of the wound bed microenvironment, and in particular the wound fluid (WF), could represent an important source of biomolecular information, and explain how the disruption of the normal healing process results in ulcer chronicity. To date, there is no single biomolecular signal that could be used to indicate the wound healing process is progressing. From our recent biochemical studies on chronic venous wound fluids assessing cytokines, chemokines, and metalloproteinases [2,4,5], we focused this study on the role of Transforming Growth Factor-β (TGF-β) isoforms and soluble endoglin (sEng), as critical inflammatory mediators [6,7] in both vascular diseases [8,9,10] and wound healing [11,12], and their production/release in different stages of healing in VLU.

In particular TGF-β1, among the first factors released in response to a tissue injury with TGF-β2, is important in regulating leucocyte and fibroblast recruitment; ECM remodeling; and keratinocyte proliferation, differentiation, and migration [13,14]. Moreover, TGF-β1 and TGF-β2 contribute to the ECM remodeling by both stimulating fibrogenesis and deposition of collagen I and II, fibronectin, vitronectin, tenascin, and proteoglycans [15], and also upregulating the synthesis of TIMPs (tissue inhibitor of metalloproteinases), finally limiting the proteolytic activity of MMPs (matrix metalloproteinases) necessary for proceeding to the granulation tissue formation in venous ulcer and enhancing wound contraction [7,16,17,18]. On the contrary, TGF-β3, the less expressed and not fully understood among the three TGF-β isoforms, acting as a TGF-β1-antagonist leads to a progressive decrease of collagen type I deposition and fibroblast differentiation and enhancing the MMP-9 expression and proteolytic activity [12].

In this network, another important mediator is endoglin (Eng, CD105), an integral glycoprotein composed by extracellular, transmembrane, and cytoplasmic domains, functioning as an auxiliary co-receptor for TGF-β type II receptor [8,9]. The extracellular domain of Eng is characterized by a sequence of arginine-glycine-aspartate (RGD motif), responsible for the inflammatory-linked leukocyte adhesion and transmigration [6]. Matrix metalloproteinase (MMP)-14 is able to cleave the extracellular domain of Eng, releasing into the circulation its soluble form (sEng) [19], which may counteract the TGF-β signaling, playing a crucial role in cardiovascular diseases [9]. The solubilization of Eng may control endothelial cell shape changes in response to hemodynamic shear stress [8,19], a crucial step in CVeD initiation and progression [1,3]. However, the high abundance of Eng on the membrane of vascular endothelial cells and activated monocytes/macrophages reflects its key roles in the vascular homeostasis and vessel inflammatory processes [6,8]. Interestingly, the abundance of membrane-bound forms of Eng has been histochemically studied during the wound healing processes, revealing a significant upregulation and involvement of Eng in dermal repair and infiltration of inflammatory cells [11,20].

Although clear evidence exists on TGF-β in CVeD [2], no data were published in literature about the presence of sEng in chronic ulcer fluids related to the different release of TGF-β isoforms during the states of wound healing of the chronic ulcer microenvironment.

In this respect, our study aimed to investigate the abundance of all the three TGF-β isoforms and sEng in wound fluid (WF) of both non-healing and healing VLU and to identify their potential interactions with the inflammatory and proteolytic mediators. Furthermore, we studied the release of TGF-beta isoforms and sEng from WF-stimulated monocytes, with and without the treatment of sulodexide (Alfa Wassermann, Bologna, Italy), a glycosaminoglycan mixture known for its antithrombotic and pro-fibrinolytic activities, as well as for the anti-inflammatory and endothelial-protective effects in CVeD [21,22,23,24,25]. Identifying differences of TGF-β isoforms and sEng in VLU WF of different healing stages provides important information on pathophysiological processes and possible therapeutic targets for VLU healing. In addition, a better understanding of how sulodexide modulates the production and release of monocyte TGF-β and sEng when exposed to WF provides clinical support in its application to the treatment of VLU and its possible role in VLU healing.

## 2. Results

### 2.1. Demographic Data

**Figure 1:**
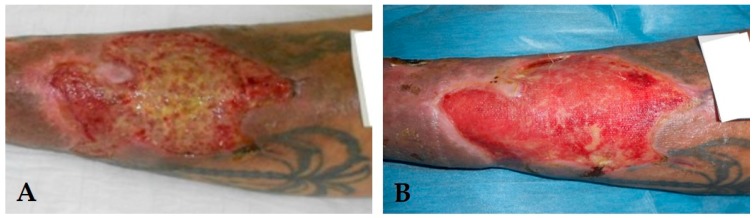
Clinical aspects of different wound healing phases in the same patient. In (**A**) a representative example of venous leg ulcer “blocked” in the inflammatory state of wound healing (inflammatory ulcer); In (**B**), the same patient underwent to conservative or surgical therapeutic procedures which turned the ulcer in granulation state (granulating ulcers).

Thirty patients with VLU were enrolled in the study. According to the state of wound healing and to the clinical observation of the ulcer tissues, WF was subdivided into two groups: inflammatory (Infl, *n* = 20) and granulating (Gran, *n* = 10) (total WFs, *n* = 30) (Figure 1).

The biological samples examined were collected from both sexes (10 males and 20 females), with a mean age of 73.7 years (range: 43–91 years), and presenting ulcers both at first episode (*n* = 11) or recurrent (*n* = 19). The mean ulcer duration was 41.6 ± 54.5 months. The average ulcer size was 10.7 cm^2^ (range 0.2–60 cm^2^). Complete demographic variables for the study population are presented in Table 1.

For what concerns demographic characteristics, we considered that patient-related factors may lead to possible bias; however, most of the epidemiological and demographic factors were similar between the two groups (except for hyperlipidemia and infection). In our work, we have considered as unit of measure the “type of wound” that the patient had at the time of wound fluid collection (commonly classified as inflammatory and granulating states) to capture the information on how the biomarkers expressed in the wound fluid may characterize each wound type.

### 2.2. Pain Scale

Patients with Infl WF had significantly increased pain scales compared to patients with Gran WF (5.3 ± 1.3 vs. 3.4 ± 0.8, respectively, *p* = 0.0004) (Table 1).

### 2.3. TGF-β Isoform and sEng Determination in Wound Fluids

**Figure 2:**
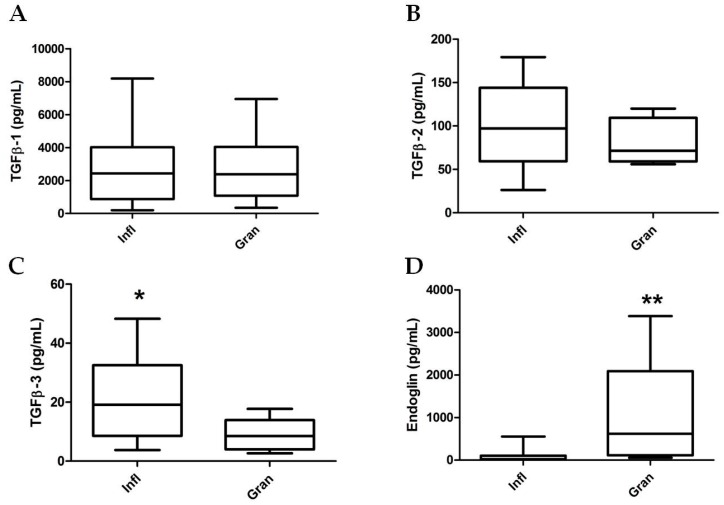
TGF-β isoforms and sEng levels in inflammatory and granulating wound fluids (*n* = 20 and *n* = 10, respectively). Statistical analyses by Mann–Whitney test. (**A**) Transforming growth factor-β-1 (TGFβ-1); (**B**) TGFβ-2; (**C**) TGFβ-3; and (**D**) soluble Endoglin concentrations in wound fluids collected from Inflammatory (Infl) and Granulating (Gran) leg ulcer state. (* = *p* < 0.05 ; ** = *p* < 0.01).

The quantitative abundance of the three TGF-β isoforms in 30 WF samples from VLU, revealed that TGF-β1 and TGF-β2 levels were not statistically different in Infl vs. Gran WF samples (*p* = 0.947 and *p* = 0.301, respectively). On the contrary, TGF-β3 concentration was significantly increased in Infl vs. Gran WF (Infl vs. Gran: 20.25 ± 3.30 vs. 9.10 ±1.74 pg/mL, respectively; *p* = 0.033) (Figure 2).

It is worth noting that a statistically significant increased concentration of sEng was found in Gran compared to Infl WF samples (Infl vs. Gran: 92.98 ± 34.5 vs. 1042.0 ± 435.2 pg/mL, respectively; *p* = 0.002) (Figure 2), suggesting that in VLU WF microenvironment a different shedding of sEng may occur during the healing process, and may have significant implications in the pathogenesis of VLU.

### 2.4. Effects of Sulodexide on WF-Stimulated THP-1 Monocytes

As assessed by trypan blue exclusion test, the cell viability of THP-1 cells treated with 5% WF was >85% and >95% for Infl and Gran, respectively.

To better clarify the involvement of monocyte/macrophage in wound healing, we treated human monocyte THP-1 cells with inflammatory stimuli induced by WF, with and without the co-treatment with sulodexide, a glycosaminoglycan mixture composed of heparin and dermatan sulfate and efficacious in the treatment of VLU [26,27].

**Figure 3:**
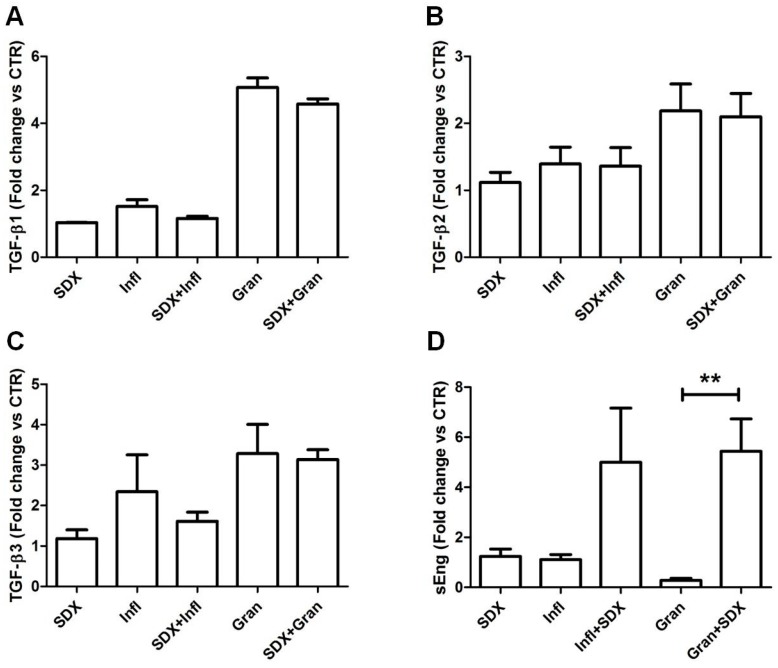
Concentrations of TGF-β isoforms (**A**: TGF-β1; **B**: TGF- β2; **C**: TGF- β3) and sEng (**D**) in culture media supernatants from WF-stimulated THP-1 cells in the presence or absence of the glycosaminoglycan sulodexide (SDX); (** = *p*<0.01). Statistical analyses with ANOVA followed by Dunns post hoc test.

As depicted in Figure 3, the treatment of monocytes with sulodexide alone did not show any significant modification in the release of TGF-β isoforms and sEng in culture media.

Although we found an increased shedding of sEng in culture media (up to three-fold) after the co-treatment of THP-1 cells with sulodexide plus WF (both Infl and Gran WF) (Figure 3D), results were statistically significant only for the THP-1 cells treated with Gran WF (ANOVA test, *p* < 0.01).

Our data suggest that only the interaction of glycosaminoglycans with biomolecules present in WF is able to induce the proteolytic shedding of sEng in culture media.

## 3. Discussion

The physiological process of wound repair requires a progressive stimulation of angiogenesis, keratinocyte and fibroblast proliferation and migration, epithelial, and endothelial basement membrane regeneration and connective tissue deposition to allow the granulation tissue development and the skin barrier restoration. This sequence of events is strictly coordinated by different cellular types (e.g., keratinocytes, fibroblasts, endothelial cells, monocytes, neutrophils, and platelets), as well as ECM components, inflammatory mediators, and proteolytic enzymes taking place in wound microenvironment [28].

In this regard, our study revealed that TGF-β3 level was significantly higher in Infl WF compared to Gran WF, whereas sEng was significantly increased in Gran WF compared to Infl WF.

Our results are in agreement with literature data suggesting that TGF-β3 isoform antagonizes the effect triggered by the other TGF-β isoforms and promotes the release of pro-inflammatory cytokines and chemokines such as TNF-α, IL-1, IL-6, and CXCL8/IL-8 [29,30].

In fact, during wound repair TGF-β1 is initially upregulated due to its chemoattractant properties and proteolytic activity induction; its levels gradually decline during the final reparative phase to promote keratinocyte and fibroblast migration and differentiation and fibrogenesis. Similarly, TGF-β2 contributes to collagen deposition and angiogenesis. TGF-β3 has a negative effect on TGF-β1 and may contribute to the ulcer chronicity also inhibiting myofibroblasts formation [31,32]. It is noteworthy that the TGF-β3 isoform has been also identified to be mainly released by neutrophils [33], a cell type found in the ulcer bed of infected VLU [34].

Even though the increasing concentration of TGF-β3 isoform during the inflammatory state seems to counteract the healing process, the higher levels of sEng observed within Gran WF may be linked to a shedding process related to the well-known upregulated proteolytic network found in WF [2,4]. In fact, searching by protease specific prediction server database (available online: https://prosper.erc.monash.edu.au/) we found that membrane-bound Eng contains putative substrate sequences for several human proteases—including calpain-1, cathepsin G and K, MMP-2, -3, and -9—expressed in WF [2,4].

The increased level of sEng in Gran WF is also in agreement with previous evidence suggesting that sEng competes for the interaction between leukocyte β1-integrin and endothelial membrane-anchored endoglin [6]. sEng likely affects the leukocyte adhesion and transmigration through the endothelium causing a reduction in the inflammatory response, and thereby favoring the granulating state of wound closure.

Moreover, sEng, antagonizing the functions of its membrane-bound form, inhibits angiogenesis, capillary tube formation, and sprouting, and increases vascular permeability [19].

In the Gran WF from VLU microenvironment, this observation may indicate a slowing down in the angiogenic activity, also induced by sEng interaction with TGF-β1, thus preventing its binding to the cell membrane receptor [8].

In addition, we observed that the glycosaminoglycan sulodexide treatment was able to significantly increase the shedding of sEng in Gran WF-stimulated monocytes. A possible mechanism explaining this observation may be linked to the presence of glycosaminoglycan-degrading enzymes in WF (e.g., heparanase), known to alter the TGF-β and Eng signaling, modulate proteolytic pathways, and alter betaglycan functions [35,36].

Our in vitro results suggested that glycosaminoglycan sulodexide, in the granulating microenvironment, may be able to stimulate the release of sEng from the membrane-bound form, thus affecting the TGF-β signaling, as well as the leukocyte-endothelium interaction.

In the process of wound healing, it is well known that fibroblasts are activated to synthesize and secrete collagen; moreover, TGF-β antagonizes the effects of inflammatory cytokines by inhibiting the release/activity of MMPs. It plays an important role in ECM metabolism in wound healing process and promotes wound healing and collagen deposition [19].

Although further studies are needed to confirm this hypothesis, our results for the first time focus attention to the “paradox of endoglin biology” [9] in WF samples during different wound healing states of VLU. However, although the present study is conceived as pilot project and exploratory concept study, we are aware that the result provided is limited by the lack of statistical power, and should be perceived as evidence for the potential existence of the molecular mechanism based on endoglin involvement in wound healing. We highlighted that, besides the well-established roles of membrane-bound endoglin in cardiovascular physiopathology, the proteolytic release of sEng in VLU microenvironment may guide the reparative mechanisms of wound closure. This result suggests a potential utility of monitoring sEng in endothelial dysfunction [37] and targeting its signaling related to transforming growth factor beta isoforms modulation [38] by glycosaminoglycan sulodexide as a promising hypothetical therapeutic approach for patients suffering of CVeD.

## 4. Materials and Methods

### 4.1. Patient Selection and Recruitment Criteria

The production of TGF-β isoforms and sEng in WF was studied in 30 patients affected by non-healing VLU and admitted to the hospital to undergo surgical debridement and skin grafting.

Patients who met the following criteria at the start of the recruitment were eligible for participating in the study: patients of any gender, older than 18 years, with primary or secondary venous disease, with chronic, non-healing VLU both at first episode and relapsing.

Written informed consent was obtained from all patients. The study was approved from the local ethics committee (both Barbantini Clinics of Lucca and University “Carlo Bo” of Urbino; protocol No. 16781, 1 July 2015) and was also in accordance with ethical standards of the Helsinki Declaration of 1975, as revised in 2000.

Exclusion criteria were as follows: age less than 18 years, pregnant or breast feeding women, presence of arterial disease, renal insufficiency, insulin-dependent diabetes mellitus, vasculitis, autoimmune disease, cortisone or immunosuppressant or hormonal therapies, previous venous surgery, or sclerotherapy.

Data on the medical history of all patients had been recorded, and clinical and duplex ultrasound venous examinations had been performed. Venous pathophysiology was identified according to the clinical aspect and confirmed with Duplex ultrasound scanning examination. Duplex ultrasound was performed in the standing position with the weight on the contralateral leg. Venous reflux was elicited by means of calf compression-release maneuver, and diagnosed when venous reflux was greater than 0.5 s in the superficial venous system and greater than 1 s in the deep venous system [39]. The presence of thrombosis was evaluated with compression ultrasounds. Chronic venous disease was classified according to the CEAP classification [40], and recruited as C6 stage, active ulcer.

### 4.2. Chronic Venous Ulcer Wound Fluid Protocol

Wound fluids were collected at the initial admission to the hospital when VLU were divided in two groups according to the clinical examination by medical doctor expertise in: (a) inflammatory (Infl, *n* = 20 patients) and (b) granulating (Gran, *n* = 10 patients) ulcers, in agreement to previously described protocols [4,5]. All patients were treated with inelastic multilayer compression ≥60 mm Hg in the supine position. Skin grafting or foam sclerotherapy were eventually performed after wound fluid sampling.

All patients underwent biopsy of the ulcer bed to perform quantitative bacterial analysis, and pain was assessed with a visual analog scale (VAS), which rates pain intensity on a scale from 0 to 10, where 0 = no pain; 1–3 = mild pain; 4–6 = moderate pain; 7–10 = severe pain [41].

WF was collected by applying cotton gauze to the ulcer bed until saturated, WF-embedded gauze was transferred in a collecting tube without additives or antiproteases, and then centrifuged at 10,000× *g*. The supernatant was stored at −80 °C until further analysis.

### 4.3. Cell Culture and Treatments

Human monocytic THP-1 (ATCC^®^ TIB-202™) cell line obtained from American Type Culture Collection (Manassas, VA, USA) was grown in standard culture conditions (RPMI 1640 supplemented with 10% heat-inactivated fetal bovine serum, 1% l-glutamine, and 1% antibiotics) and maintained at 37 °C in humidified air with 5% CO_2_. The experiments were performed in serum-free conditions to avoid the recovery of endogenous bovine serum TGF-β and sEng.

THP-1 cells were seeded at 1,500,000/mL and stimulated with WF collected from patients with Infl or Gran VLU. For this purpose, aliquots (with the same volume) from 10 randomly selected samples of WF from each group (*n* = 10 Infl and *n* = 10 Gran WF) were pooled. After filtration with 0.45 μm tissue culture filter unit to remove large cell debris and bacteria, both the pooled Infl and Gran WF were immediately diluted in the Roswell Park Memorial Institute formulation of serum-free culture media (RPMI 1640) to a final concentration of 5% *v*/*v*, where treated cells were grown for 24 h, in the presence or absence of sulodexide co-treatment to a final dose of 0.12 LSU/mL. Cell viability was assessed by trypan blue exclusion test. Each experiment on serum-free conditioned medium was performed in triplicate in at least two independent experiments. Sulodexide treated and untreated THP-1 monocytes exposed to Infl and Gran WF were analyzed for the abundance of TGF-β isoforms and sEng using magnetic multiplex immunoassay.

### 4.4. Magnetic Multiplex Immunoassay

TGF-β isoform and sEng levels in WF and serum free culture media were determined through the 3-plex panel of Pro™ Human TGF-β 3-plex Assay (including TGF-β1, TGF-β2, and TGF-β3) and the single-plex Endoglin, as a part of the Pro™ Human Cancer Panel 2 Assay, a multiplex suspension immunomagnetic assays, based on the use of fluorescently dyed magnetic beads covalently conjugated with monoclonal antibodies specific for the target proteins, according to the manufacturer’s instructions (BioPlex, Bio-Rad Labs, Hercules, CA, USA).

Levels of all analytes were determined using a Bio-Plex 200 array reader, based on Luminex X-Map Technology (Bio-Rad Labs, Hercules, CA, USA) that detects and quantifies multiple targets in a 96-well plate with a single small fluid volume. The protein concentrations (expressed as pg/mL) were calculated through a standard curve. Although the commercially available kit of TGF-β isoforms and sEng allow to analyze several biological fluids other than plasma, to exclude in assays the possible WF “matrix” artifacts caused by possible interference substances, we serially diluted randomly selected WF samples, reanalyzing them for the response linearity. According to the manufacturer’s data, the lower detection limit was 3.9 pg/mL (TGF-β1), 1.9 pg/mL (TGF-β2), 0.5 pg/mL (TGF-β3), and 1.0 pg/mL (sEng) while the mean inter-assay variability was 7.4% for TGF-β isoforms and 9.6% for sEng.

### 4.5. Chemicals

Glycosaminoglycan sulodexide was provided by Alfa Wassermann (Milan, Italy). All chemicals of reagent grade were obtained from Sigma (Milan, Italy), whereas the sterile compounds for cell culture were from JET BIOFIL Bio-filtration Products Co (Guangzhou, China), and chemicals and reagents for cell culture were from Carlo Erba Reagents S.r.l. (Milan, Italy).

### 4.6. Statistical Analysis

Each variable was expressed as the mean ± standard error of the mean, unless otherwise specified. Statistical analyses were carried out through Fisher Exact test, Mann–Whitney, one-way analysis of variance ANOVA with repeated measures, followed by Dunn’s post hoc test according to variable characteristics. The exact statistical tests were reported and described in the Tables and Figures. All statistical tests were two-tailed, and significance was set at *p* < 0.05. Data and graphs were analyzed with Prism software for Windows 7, version 3.1 (Graph-Pad, San Diego, CA, USA). We defined this study as pilot preclinical study, therefore we did not determine a power calculation. Accordingly, the results can only be labeled as a pilot project with an exploratory concept study characteristic.

## 5. Conclusions

Venous leg ulcer (VLU) is the final complication of the chronic venous disease (CVeD), a common lower extremity vascular pathology of great medical and socioeconomic impact, affecting a large part of the population worldwide.

Transforming Growth Factor-β (TGF-β) and Endoglin (Eng), are mediators in inflammation, fibrosis, and wound healing. Eng is a co-receptor for TGF-β type-II receptors. Extracellular domain of Eng is cleaved forming soluble Eng (sEng), which antagonize TGF-β signaling.

Our study revealed that TGF-β3 level was significantly higher in Infl WF compared to Gran WF, whereas sEng was significantly increased in Gran WF compared to Infl WF. Our results are in agreement with literature data suggesting that TGF-β3 isoform antagonizes their effect and promotes the release of pro-inflammatory cytokines and chemokines such us TNF-α, IL-1, IL-6, and CXCL8/IL-8. The increase in TGF-β3 found in Infl WF is consistent with its negative effects on wound healing, while the increased levels of sEng in Gran WF likely affects the leukocyte adhesion/transmigration through the endothelium reducing the inflammatory response and favoring wound healing.

Our in vitro results suggested that glycosaminoglycan sulodexide, in the granulating microenvironment, may be able to stimulate the release of sEng from the membrane-bound form, thus affecting the TGF-β signaling, as well as the leukocyte-endothelium interaction. The proteolytic release of sEng in VLU microenvironment may guide the reparative mechanisms of wound closure, suggesting a potential utility in monitoring sEng in endothelial dysfunction and targeting sEng signaling by glycosaminoglycan sulodexide as a promising therapeutic approach for patients suffering from CVeD.

## Figures and Tables

**Table 1:** Demographic and clinical characteristics.

Characteristic	Inflammatory	Granulating	*p* Value
Number, *n* (%)	20 (67)	10 (33)	
Age range, years	43–91	65–85	
Mean age (±SD), years	71.4 ± 14.4	77.9 ± 6.6	0.370
Sex			0.231
Male, *n* (%)	5 (25)	5 (50)	
Female, *n* (%)	15 (75)	5 (50)	
Comorbidities			
Diabetes, *n* (%)	7 (35)	2 (20)	0.675
Hypertension, *n* (%)	11 (55)	9 (90)	0.101
Hyperlipidemia, *n* (%)	12 (60)	1 (10)	0.017
Smoking, *n* (%)	2 (10)	0 (0)	0.540
Rheumatic disease, *n* (%)	2 (10)	0 (0)	0.540
Ulcer History			0.702
Primary	8 (40)	3 (30)	
Recurrent	12 (60)	7 (70)	
Infection	14 (70)	0 (0)	<0.001
Duration, months, mean ± SD	51.4 ± 63.9	22.2 ± 18.3	0.481
Surface area, cm^2^, mean ± SD	12.7 ± 17.9	6.7 ± 4.2	0.947
VAS * score, mean ± SD	5.3 ± 1.3	3.4 ± 0.8	<0.001

* VAS: visual analog scale; Statistical tests: Fisher exact test for categorical variables, and Mann–Whitney test for continuous variables (i.e., age, duration, area and VAS).

## Abbreviations

CEAP	Clinical, etiological, anatomical, and pathophysiological classification
CVeD	Chronic venous disease
CVI	Chronic venous insufficiency
ECM	Extracellular matrix
IL	Interleukin
MMP	Matrix metalloproteinase
TGF-β	Transforming growth factor-β
VLU	Venous leg ulcer
WF	Wound fluid
